# Arthroscopic Repair of Articular Surface Partial-Thickness Rotator Cuff Tears: Transtendon Technique versus Repair after Completion of the Tear—A Meta-Analysis

**DOI:** 10.1155/2016/7468054

**Published:** 2016-07-04

**Authors:** Yohei Ono, Jarret M. Woodmass, Aaron J. Bois, Richard S. Boorman, Gail M. Thornton, Ian K. Y. Lo

**Affiliations:** ^1^Department of Surgery, Section of Orthopaedic Surgery, McCaig Institute for Bone and Joint Health, University of Calgary, Calgary, AB, Canada T2N 4Z6; ^2^Department of Orthopaedic Surgery, Nagoya University Graduate School of Medicine, Nagoya 466-8560, Japan; ^3^Department of Orthopaedics, University of British Columbia, Vancouver, BC, Canada V5Z 1M9

## Abstract

Articular surface partial-thickness rotator cuff tears (PTRCTs) are commonly repaired using two different surgical techniques: transtendon repair or repair after completion of the tear. Although a number of studies have demonstrated excellent clinical outcomes, it is unclear which technique may provide superior clinical outcomes and tendon healing. The purpose was to evaluate and compare the clinical outcomes following arthroscopic repair of articular surface PTRCT using a transtendon technique or completion of the tear. A systematic review of the literature was performed following PRISMA guidelines and checklist. The objective outcome measures evaluated in this study were the Constant Score, American Shoulder and Elbow Surgeons score, Visual Analogue Scale, physical examination, and complications. Three studies met our criteria. All were prospective randomized comparative studies with level II evidence and published from 2012 to 2013. A total of 182 shoulders (mean age 53.7 years; mean follow-up 40.5 months) were analyzed as part of this study. Both procedures provided excellent clinical outcomes with no significant difference in Constant Score and other measures between the procedures. Both procedures demonstrated improved clinical outcomes. However, there were no significant differences between each technique. Further studies are required to determine the long-term outcome of each technique.

## 1. Introduction

Rotator cuff tears, both full thickness and partial-thickness rotator cuff tears (PTRCTs), are common cause of pain and dysfunction in the adult shoulder [1]. Due to age-related degeneration, rotator cuff tears may present in various locations, sizes, shapes, and severity. Perhaps paradoxically, the degree of rotator cuff tearing does not necessarily correlate with the severity of symptoms and in many instances PTRCTs may be more painful than full thickness tears [2, 3].

The classification of PTRCTs was described by Ellman and based on the depth of the tear [grade I: less than 3 mm (<25% of thickness), grade II: 3 to 6 mm (25–50% of thickness), grade III: >6 mm (>50% of thickness)], and their anatomical location (articular surface, bursal surface, and interstitial) [4]. While many patients with smaller PTRCT can improve clinically with conservative treatment modalities (e.g., medications, physiotherapy, and injections), surgical repair may be indicated in patients with larger tears or those who have failed nonsurgical treatment [5, 6]. While there are a number of factors to consider including age, activity level, vocation, sports participation, chronicity of symptoms, and associated pathology, currently, surgical reattachment of the tendon is usually indicated for the PTRCTs involving 50% or more of the tendon thickness [6–8].

While a number of surgical procedures have been described for the repair of articular surface PTRCTs, the most commonly reported procedures have been the transtendon repair technique and formal repair after completion of the PTRCT. Traditionally, the technique of tendon repair after completion of the PTRCT has been performed. In this technique the residual intact rotator cuff tendon is taken down converting the PTRCT to a full thickness tear. This technique provides better visualization and allows standard rotator cuff repair techniques to be utilized for repair. This procedure has also been shown to provide satisfactory clinical outcomes [9–13]. However, concerns with respect to the resection of the intact tendon have led to the development of the transtendon technique.

Over the last two decades, the transtendon repair has increased in popularity with reported successful clinical outcomes [14–20]. In this technique the intact bursal surface of the rotator cuff tendon is preserved and is maintained attached to greater tuberosity. The theoretical advantage of this procedure is better healing with improved biological and biomechanical repair characteristics. However, the procedure is more complex and is hindered by a restricted working space and visualization. Furthermore, some authors have raised concerns over damaging the intact tendon during transtendon anchor insertion and overtensioning the repair [20, 21].

Although a number of biomechanical studies have demonstrated superior fixation strength of a transtendon repair when compared to completion of the tear with subsequent repair, few studies have directly compared the two procedures [21, 22]. Furthermore the majority of studies evaluating these two techniques have been case series of one technique with no comparison group. Recently, however, a number of prospective, randomized trials have been published comparing the transtendon repair with completion of the tear with subsequent repair [23–25]. Therefore, the purpose of the study was to evaluate and compare the clinical outcomes following arthroscopic repair of articular-sided PTRCT by performing a meta-analysis of the current high quality studies.

## 2. Materials and Methods

### 2.1. Systematic Review

**Figure 1:**
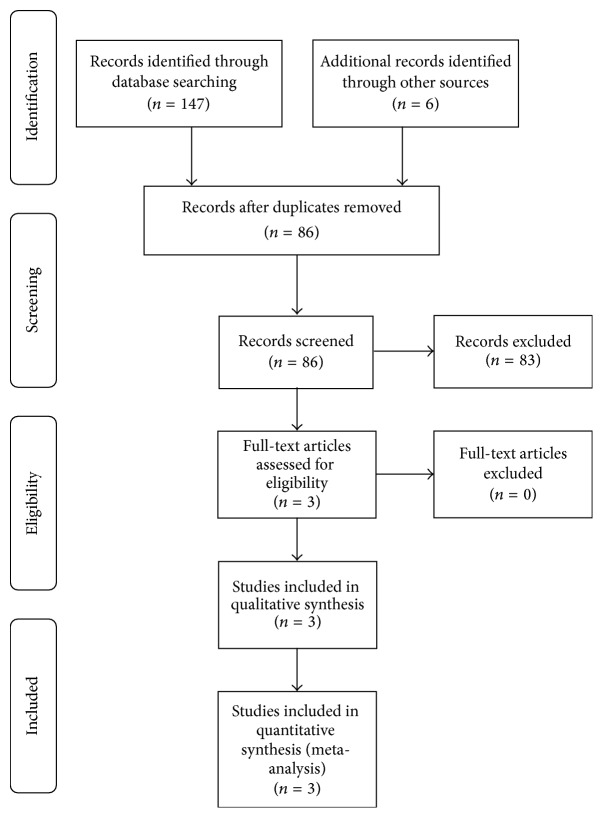
Systemic review algorithm using PRISMA guidelines.

We performed a meta-analysis of the literature using the Preferred Reporting Items for Systemic Reviews and Meta-Analyses (PRISMA) guidelines and checklist. The search algorithm according to the PRISMA guidelines is shown in Figure 1. A thorough literature search of the following databases was conducted: PubMed, MEDLINE, CINAHL, and Cochrane Data Base. The search terms used in various combinations included “shoulder”, “arthroscopy”, “rotator cuff”, “partial thickness tear”, “articular side”, “PASTA”, “repair”, “transtendon”, “completion”, “conversion”, and “prospective.” Our inclusion criteria included English language studies, anchor based arthroscopic rotator cuff repair surgery outcomes, supraspinatus and/or infraspinatus tendon repairs, techniques of transtendon cuff repair, and cuff repair after completion of the tear. The exclusion criteria included non-English language studies, retrospective studies, nonrandomized studies, full thickness rotator cuff repairs, concomitant instability surgeries, nonanchor based arthroscopic cuff repairs (transosseous, tacks), acute fractures, systemic reviews/meta-analyses, letters to the editor, basic science studies, biomechanical studies, surgical technique studies, meeting abstracts/proceedings, and studies of duplicate patient populations.

To ensure only modern surgical techniques were included in the analysis, each study was also evaluated specifically for operative technique. Only arthroscopic rotator cuff repairs that utilized suture fixation of the tendon to bone using suture anchors were included in the study. Using the levels of evidence as outlined by the Oxford Centre for Evidence Based Medicine, level I or II studies that fit the above inclusion criteria were included in this study. Three independent investigators conducted the search separately, each reviewing the abstract of each publication, followed by extracting the data from each relevant article. The final literature search was conducted on October 17, 2014. In addition, we cross-referenced all references of included studies to avoid omitting relevant studies not included in original search. In the event there was disagreement regarding the inclusion of a study, the senior author ultimately made the final decision. For studies using duplicate patient populations, only the most recent publication was used for analysis.

### 2.2. Quality Assessment

We used the Coleman Methodology Score (CMS) to assess the quality of the studies. The CMS is a 15-item checklist that produces a scaled 100-point score; a score between 85 and 100 is considered excellent, 70–84 good, 55–69 fair, and less than 55 poor. A perfect score indicates a study that avoids chance, bias, and confounding variables. The CMS was performed by 2 independent reviewers (1 orthopaedic resident and 1 shoulder fellow), with all results confirmed by the senior author. The CMS has previously been used in other orthopedic and sport medicine research and is considered a strong quality assessment tool for studies of this nature [26–28].

### 2.3. Outcome Measures

The objective outcome measures evaluated in this study included the Constant Score, American Shoulder and Elbow Surgeons (ASES) score, Visual Analogue Scale (VAS), range of motion, tendon healing on MRIs, and postoperative complications. Three reviewers evaluated the literature separately and any discrepancies were reevaluated and resolved by consensus.

### 2.4. Statistical Analysis

The data consist of three prospective randomized trials comparing two surgical treatments. The outcome was difference in mean improvement in Constant Score between completion group and transtendon group. The three mean study differences were provided, but within-study standard deviations were only available for one study; therefore the analysis was a fixed effects model weighted by sample size.

## 3. Results

### 3.1. Literature Search

The literature search together with cross-referencing yielded 153 articles. Of these 153 articles, 67 abstracts failed to meet our inclusion/exclusion criteria and were subsequently excluded. An additional 83 full-text articles were excluded due to insufficient details (Figure 1). In total 3 studies met our inclusion criteria and were included in our analysis. All included articles were prospective randomized comparative studies classified as level II evidence. These 3 studies were all published between 2012 and 2013 (Table 1).

### 3.2. Methodology Assessment

The mean CMS was 91.3. The highest rating given was 100, while the lowest was 87. According to the classification system, all the 3 studies were deemed excellent (Table 1).

### 3.3. Demographics

A total of 182 shoulder repairs were analyzed with the weighted mean age of 53.7 years. The number of repairs included for analysis was 93 for transtendon repair and 89 for repair after completion of tear, with the weighted mean patient age being 54.9 years and 52.4 years, respectively. The weighted mean follow-up period after surgery was 40.5 months (Table 2). Similar postoperative rehabilitation protocols were utilized, with the shoulder immobilized with a small abduction pillow for 4 weeks, followed by range of motion exercises. Resistance exercises and strengthening were begun at 3 months progressing to full function by 4 to 6 months.

### 3.4. Function and Pain Scores

All the 3 studies evaluated clinical outcome measures at final follow-up (between 24 and 38 months). Constant Score was the only outcome which was evaluated in all the studies and other measures such as ASES, VAS, range of motion, healing on imaging, and complications were not determined consistently. Therefore, the Constant Score was used as the primary measure for statistical analysis. All 3 studies showed no baseline difference between the two procedures and statistically significant improvement in the Constant Score from preoperatively to postoperatively at final follow-up. The 3 mean study differences in improvement of Constant Score between completion group and transtendon group were 1.0, 3.9, and −1.9. The combined effect size was estimated as 1.419 (95% CI −1.839, 4.676), *p* = 0.483. There was no evidence of a difference between groups in mean improvement in Constant Score.

ASES and VAS were evaluated in 2 of the 3 studies with statistically significant improvement from preoperatively to postoperatively at final follow-up. Both procedures provided generally excellent clinical outcomes and none of these studies show any significant difference in these clinical outcome scores between the two procedures (Table 3). However, interestingly, one of the 3 studies evaluated these 3 scores at earlier stages postoperatively in addition to the final follow-up, showing significantly faster recovery at 3 months in completion group than in transtendon group (Table 5).

### 3.5. Range of Motion

Range of motion was examined pre- and postoperatively in 2 studies. Patients in both groups were significantly improved for forward elevation, external rotation, and internal rotation at the final follow-up. None of these studies show any significant difference in range of motion between the two procedures (Table 4).

### 3.6. Tendon Healing

Healing of the repairs was examined on postoperative MRIs by a blinded musculoskeletal radiologist in 2 studies. MRIs were performed at 6 months by Shin [25] and at final follow-up (38 months) by Franceschi et al. [23]. In the transtendon group, all repairs except one were considered healed by MRI. However, there were three repairs in the tear completion group which were considered not healed or retorn. The healing rate was 98.2% in transtendon group and 93.9% in tear completion group (Table 6).

### 3.7. Complications

Adhesive capsulitis was the only complication reported in 2 studies. During follow-up, 6 patients (10.9%) in transtendon repairs and 5 patients (10.2%) in repairs after completion of tear developed adhesive capsulitis, where 1 patient (1.8%) and 2 patients (4.1%) required arthroscopic capsular release, respectively. However, all of those patients were successfully treated either conservatively or operatively with good functional outcomes (Table 7).

## 4. Discussion

Clinical outcomes after surgical repair of PTRCTs have been reported to be successful in both short to midterm reports [9, 10, 12–14, 18, 19]. However, the technique utilized to repair the PTRCT remains controversial.

Although surgical repair for PTRCTs has been reported in the literature, until recently there were few high quality studies comparing these 2 types of procedures. From 2012 to 2013, three prospective, randomized comparative studies have been published with level II evidence. All of these studies met our inclusion criteria and no other studies were found to be eligible for our analysis.

In the present study, clinical outcomes, including function and pain scores and range of motion, of both procedures were significantly improved postoperatively. Healing of the repaired tendons was evaluated postoperatively on MRI in 2 studies, with only 4 cases (3.7%) reported to be unhealed but with comparable clinical outcomes to those of the other patients with healed repairs. Adhesive capsulitis was the only complication reported and 3 patients (2.8%) required another surgery for capsular release although those cases did clinically well at final follow-up. Thus, either of these 2 procedures appears to provide reasonably successful clinical outcomes. When comparing the 2 procedures, neither study demonstrated any significant differences in those outcome measures during the follow-up of 40.5 months. Furthermore, collectively, the current study demonstrated no significant difference between groups in mean improvement in Constant score, which was used a primary measure in all the studies.

The Constant Score is the most widely used scoring instrument to assess shoulder function, particularly for rotator cuff disease [26–28]. The minimal clinically important difference (MCID) of Constant for rotator cuff surgery has been reported to be 10.4 by Kukkonen et al. [28] and 18 by Henseler et al. [27]. Given the estimated combined effect size of 1.419 in Constant Score in our study, the difference between the two procedures was not only not statistically significant but also likely not clinically important. Furthermore, this suggests that even if further high level studies were performed and more patients were included in the study although a statistical difference may be able to be demonstrated between the two groups it would likely be clinically unimportant as well. Given the results of our meta-analysis, both procedures can provide satisfactory short-term to midterm outcomes and therefore either technique may be performed when faced with a PTRCT.

Therefore while the transtendon repair has the theoretical advantage of preserving the intact bursal surface of the rotator cuff tendon with superior mechanical properties, this does not appear to translate into a superior clinical outcome in the short-term or midterm. Some surgeons believe that a transtendon repair may have higher incidence of postoperative stiffness or adhesive capsulitis [29]. Interestingly, Shin demonstrated slower recovery in clinical outcome measures at very early stages (i.e., 3 months) following transtendon repair. However, there was no difference either in the rates of postoperative adhesive capsulitis or in the clinical outcomes at final follow-up in this study.

On the other hand, repair after completion of tear is a relatively easier procedure where standard rotator cuff repair techniques may be utilized. Once the decision is made to complete the tear, removing the residual tendon to create a full thickness tear is a simple and expedient process. Interestingly, the intact tendon has been shown to demonstrate histopathologic changes consistent with degeneration supporting the removal of the residual intact tendon [30]. However, there is a concern with potential higher retear rates due to the potentially poor tendon healing after taking down the rotator cuff tendon [19]. In the present study, tendon healing on MRI was not significantly different between the groups and the retear rate was much lower than most reported rates after rotator cuff repairs ranging from 10 to 90% [31]. Both repair procedures result in healing of the tendon at a relatively high rate. Therefore, it appears that completing the PTRCT did not increase the retear rate, and the theoretical biomechanical advantages of preserving the tendon did not statistically improve healing as well. This may be related to the fact that PTRCTs (as opposed to large full thickness rotator cuff tears) already have an intrinsically good healing potential. Therefore PTRCTs may heal whatever technique is utilized. Recently, Kim et al. have reported their randomized prospective study comparing in situ repair and tear completion for PTRCTs [32]. This study was excluded from our meta-analysis since both articular- and bursal-sided tears were included and clinical outcomes for each tear type were not provided separately. However, similar to the 3 prospective trials analyzed in our study, no difference between the 2 procedures was demonstrated in any of the outcome measures, including the Constant Score. In addition there was no significant different in the retear rate of articular-sided tears repaired with either technique. However, they did demonstrate a significantly higher retear rate in bursal-sided tears treated with tear completion when compared to an in situ repair. The authors proposed that this was secondary to less ability of the bursal tissue to heal and thus the support of the remaining articular tissue may be necessary for anatomic healing. This may indicate that the present results of articular surface PTRCTs may not be applicable to the treatment of bursal-sided PTRCTs.

Due to paucity of literature comparing the transtendon procedure with the formal repair after completion of tear, only 3 studies were available and included in the current study. The Constant Score was the only measure evaluated in all the 3 studies and other measures were not determined consistently. However, all of these 3 studies are randomized prospective studies with level II evidence; therefore, we believe the information provided is sufficient to perform a meta-analysis. Since the two techniques that we compared have a relatively short history of usage and the 3 studies evaluated have only been recently published, the follow-up period is only 3 to 4 years. Therefore, there is limited information on the long-term clinical outcome of each technique. In addition, further studies evaluating each technique are still required to determine (1) early recovery, (2) cost effectiveness (number of anchors, procedure time, and learning curve), (3) retear rate, (4) tear progression, or (5) the development or arthritis.

## 5. Conclusions

At short-term to midterm follow-up, both procedures lead to improvements in clinical outcome with a low complication rate and a high rate of healing on MRI. No significant difference in clinical outcome was demonstrated between these two procedures at final follow-up. Further studies are required to determine the effect of each procedure on early postoperative recovery, long-term clinical outcome, and healing.

## Figures and Tables

**Table 1:** Study details.

Study	Authors	Journal	Study type	Level of evidence	Coleman Score	Years of patient enrollment
Articular-sided rotator cuff tears: which is the best repair? A three-year prospective randomised controlled trial	Franceschi et al. (2013) [23]	International Orthopaedics (SICOT)	Prospective randomized	II	87	2007–2009
Deep partial rotator cuff tear: transtendon repair or tear completion and repair? A randomized clinical trial	Castagna et al. (2015) [24]	Knee Surgery, Sports Traumatology, Arthroscopy	Prospective randomized	II	100	2006–2009
A comparison of 2 repair techniques for partial-thickness articular-sided rotator cuff tears	Shin (2012) [25]	Arthroscopy	Prospective randomized	II	87	2006–2008

**Table 2:** Patient demographics.

	Number of repairs	Mean age of patient (years)	Mean length of follow-up (months)
Franceschi et al. [23]	60 (32/28)	56.5 (57.3/55.6)	38.5
Castagna et al. [24]	74 (37/37)	50.5 (54/47)	>48
Shin [25]	48 (24/24)	54.0 (53/57)	31.3

Total (transtendon/completion of tear).

**Table 3:** Study outcomes.

	Procedure	Constant	ASES	VAS
Franceschi et al. [23]	Transtendon completion	44 45	45 43	(—) (—)
Castagna et al. [24]	Transtendon completion	25.1 ± 5.8 29.0 ± 6.2	(—) (—)	3.4 ± 1.2 3.6 ± 1.7
Shin [25]	Transtendon completion	30.0 28.1	38.3 37.0	4.1 4.2

Results are shown as improvement from pre-op to final follow-up.

**Table 4:** Range of motion.

	Procedure	FE	ER	IR
Franceschi et al. [23]	Transtendon completion	38.2 39.8	14.2 10.8	L3–S1 to T8–T10 L3–S1 to T8–T10
Shin [25]	Transtendon completion	26.0 33.7	15.5 20.5	L3 to T12-L1 L3 to T12-L1

Results are shown as improvement from pre-op to final follow-up.

**Table 5:** Short-term recovery.

		Procedure	Pre-op	3 M	6 M	Final follow-up
	Constant	Transtendon	54.8 ± 2.6	57.9 ± 2.9^*∗*^	72.7 ± 3.4	84.8 ± 2.7
		completion	59.0 ± 3.9	70.8 ± 3.3	80.9 ± 2.2	87.1 ± 2.4
Shin [25]	ASES	Transtendon	50.8 ± 4.3	54.9 ± 3.7^*∗*^	79.6 ± 2.5	89.1 ± 2.1
		completion	49.2 ± 4.2	64.6 ± 3.2	78.0 ± 3.4	86.2 ± 3.2
	VAS	Transtendon	5.5 ± 0.6	5.9 ± 0.4^*∗*^	2.4 ± 0.4	1.4 ± 0.4
		completion	5.3 ± 0.5	2.8 ± 0.5	1.7 ± 0.4	1.1 ± 0.2

^*∗*^Significant difference compared with completion group (*p* < 0.05).

**Table 6:** Tendon healing on MRI.

	Procedure	*N*	Healed	Not healed
Franceschi et al. [23]	Transtendon completion	32 28	31 (96.9%) 27 (96.4%)	1 (3.1%) 1 (3.6%)
Shin [25]	Transtendon completion	24 24	24 (100%) 22 (91.7%)	0 (0%) 2 (8.3%)

**Table 7:** Postoperative complication.

	Procedure	*N*	Adhesive capsulitis	Required surgery
Franceschi et al. [23]	Transtendon completion	32 28	3 (9.4%) 3 (10.7%)	0 (0%) 2 (7.1%)
Shin [25]	Transtendon completion	24 24	3 (12.5%) 2 (8.3%)	1 (4.2%) 0 (0%)

## Abbreviations

PTRCT: Partial-thickness rotator cuff tear
PASTA: Partial Articular Surface Tendon Avulsion
PRISMA: Preferred Reporting Items for Systemic Reviews and Meta-Analyses
CMS: Coleman Methodology Score
ASES: American Shoulder and Elbow Surgeons
VAS: Visual Analogue Scale.
